# Supplementary material for: Unequal Access and Use of Health Care Services among Settled Immigrants, Recent Immigrants, and Locals: A Comparative Analysis of a Nationally Representative Survey in Chile
Source: Int J Environ Res Public Health. 2022 Dec 31;20(1):741. doi: 10.3390/ijerph20010741 (PMC9819662; doi:10.3390/ijerph20010741)
Supplement: Supplementary file 1 [file ijerph-20-00741-s001.zip › ijerph-2112394-supplementary.pdf]

Supplementary Table S1 The trend of age and gender each year

	2011		2013		2015		2017		Trends
	%	IC95%	%	IC95%	%	IC95%	%	IC95%	(2011-2017)
Chilean-born	n = 16.577.539		n = 16.689.377		n = 16.970.061		n = 16.843.471		
**Men**	**47.6%**	(47.2 - 48.0)	**47.4%**	(47.0 - 47.7)	**47.3%**	(47.1 - 47.5)	**47.5%**	(47.2 - 47.7)	
**Women**	**52.4%**	(52.0 - 52.8)	**52.6%**	(52.3 - 53.0)	**52.7%**	(52.5 - 53.0)	**52.5%**	(52.3 - 52.8)	
Settled migrants	n = 157.127		n = 214.117		n = 271.783		n = 296.122		
**Men**	**42.1%**	(38.2 - 46.2)	**43.3%**	(39.1 - 47.9)	**47.8%**	(44.8 - 50.8)	**44.6%**	(42.1 - 47.2)	
**Women**	**57.9%**	(53.8 - 61.8)	**56.7%**	(52.4 - 60.9)	**52.2%**	(49.2 - 55.2)	**55.4%**	(52.8 - 57.9)	
Emerging migrants	n = 27.867		n = 62.578		n = 108.395		n = 390.488		
**Men**	**47.1%**	(64 - 58.1)	**48.9%**	(44.8 - 53.0)	**48.4%**	(45.0 - 51.8)	**51.0%**	(47.7 - 54.4)	
**Women**	**52.9%**	(41.9 - 63.6)	**51.1%**	(47.0 - 55.2)	**51.6%**	(48.2 - 55.0)	**49.0%**	(45.6 - 52.3)	
Migrants from other countries	n = 58.884		n = 77.886		n = 85.141		n = 90.797		
**Men**	**50.1%**	(43.0 - 57.3)	**46.3%**	(34.4 - 58.7)	**48.5%**	(43.5 - 53.5)	**51.2%**	(47.0 - 55.4)	
**Women**	**49.9%**	(42.7 - 57.0)	**53.7%**	(41.3 - 65.6)	**51.5%**	(46.5 - 56.5)	**48.8%**	(44.6 - 53.0)	
Chilean-born									
**0–18**	**28.3%**	(27.8 - 28.9)	**27.6%**	(27.2 - 27.9)	**26.9%**	(26.6 - 27.2)	**25.3%**	(24.9 - 25.7)	
**19–30**	**20.0%**	(19.5 - 20.5)	**19.2%**	(18.9 - 19.5)	**19.2%**	(18.9 - 19.7)	**18.7%**	(18.4 - 19.0)	
**31–65**	**41.4%**	(41.0 - 41.9)	**42.2%**	(41.8 - 42.6)	**42.1%**	(41.8 - 42.4)	**42.8%**	(42.5 - 43.1)	
**66 o more**	**10.3%**	(9.8 - 10.7)	**11.0%**	(10.7 - 11.4)	**11.7%**	(11.4 - 12.0)	**13.1%**	(12.8 - 13.5)	
Settled migrants									
**0–18**	**19.9%**	(15.9 - 24.7)	**16.8%**	(14.3 - 19.6)	**17.0%**	(14.5 - 19.7)	**15.5%**	(13.4 - 17.9)	
**19–30**	**34.1%**	(29.0 - 39.6)	**31.7%**	(27.9 - 35.7)	**33.1%**	(29.3 - 37.1)	**29.0%**	(26.1 - 32.2)	
**31–65**	**42.6%**	(36.6 - 48.8)	**47.3%**	(43.0 - 51.7)	**47.5%**	(43.3 - 51.7)	**52.4%**	(48.5 - 56.3)	
**66 o more**	**3.4%**	(2.3 - 4.9)	**4.2%**	(3.1 - 5.8)	**2.5%**	(1.9 - 3.3)	**3.0%**	(2.2 - 4.1)	
Emerging migrants									
**0–18**	**17.9%**	(13.0 - 24.2)	**13.8%**	(10.4 - 18.2)	**21.1%**	(17.4 - 25.2)	**16.4%**	(14.5- 18.4)	
**19–30**	**39.6%**	(31.5 - 48.2)	**32.7%**	(26.8 - 39.1)	**30.1%**	(25.0 - 35.9)	**44.9%**	(39.6 - 50.4)	
**31–65**	**42.1%**	(33.9 - 50.8)	**52.7%**	(47.1 - 58.2)	**47.3%**	(43.9 - 50.8)	**37.9%**	(33.1 - 43.0)	
**66 o more**	**0.4%**	(0.1 - 1.6)	**0.8%**	(0.2 - 2.7)	**1.5%**	(0.8 - 2.9)	**0.7%**	(0.4 - 1.6)	
Migrants from other countries									
**0–18**	**22.7%**	(15.9 - 31.4)	**25.1%**	(11.6 - 46.0)	**18.8%**	(15.5 - 22.6)	**12.6%**	(9.7 - 16.3)	
**19–30**	**24.6%**	(19.0 - 31.3)	**19.3%**	(14.0 - 26.0)	**23.4%**	(18.3 - 29.3)	**25.8%**	(21.1 - 31.1)	
**31–65**	**42.9%**	(34.2 - 52.0)	**45.4%**	(33.8 - 57.6)	**49.3%**	(43.7 - 55.0)	**50.7%**	(45.7 - 55.7)	
**66 o more**	**9.7%**	(6.5 - 14.3)	**10.2%**	(6.9 - 14.9)	**8.5%**	(6.0 - 11.9)	**10.9%**	(8.6 - 13.7)	

Supplementary table S2. Challenges of uninsured migrants and those without AUGE-GES treatment coverage and non-consultation in case of illness or accident.

	Uninsured migrants											
	Total migrant population				Settled migrants				Emerging migrants			
	%	IC95%	n		%	IC95%	n		%	IC95%	n	
2018 - 2017	39,84%	(29,52% - 51,16%)	70153	p-value<0,000	53,45%	(40,47% - 65,98%)	12447	p-value<0,000	36,85%	(0.2496% - 0.5060%)	52658	p-value<0,000
2015 - 2016	9,98%	(7,40% - .13,31%)	18624		15,22%	(10,80% - 21,01%)	6653		7,84%	(0.0492% - 0.1226%)	10522	
2013 - 2014	11,27%	(8,30% - 15,13%)	8253		9,25%	(6,06% - 13,86%)	3072		14,11%	(0.0778% - 0.2425%)	4398	
2011 - 2012	8,51%	(4,88% - 14,43%)	5465		7,80%	(4,52% - 13,12%)	2011		9,77%	(0.0385% - 0.2267%)	3081	
2010 - 2005	8,44%	(4,66% - 14,80%)	6449		5,75%	(3,47% - 9,38%)	3149		7,69%	(0.0408% - 0.1403%)	897	
2004 - 2000	4,98%	(3,13% - 7,85%)	1622		5,97%	(3,62% - 9,70%)	1512		2,72%	(0.0036% - 0.1783%)	41	
Pior to2000	05,3%	(3,58% - 7,77%)	2637		5,48%	(3,50% - 8,50%)	1732		8,14%	(0.0207% - 0.2704%)	234	
NR	8,29%	(5,97% - 11,39%)	9810		7,42%	(5,31% - 10,26%)	4336		9,92%	(0.0491% - 0.1901%)	3430	
	**Migrants without complementary health insurance**											
	Total migrant population				Settled migrants				Emerging migrants			
2018 - 2017	91,47%	(86,31% - 94,80%)	161064	p-value: 0,0002	88,87%	(77,44% - 94,89%)	20.694	p-value<0,0869	92,49%	(86,03% - 96,10%)	132159	p-value: 0,0158
2015 - 2016	78,58%	(69,61% - 85,46%)	146715		89,84%	(81,62% - 94,63%)	39.281		76,19%	(63,87% - 85,27%)	102300	
2013 - 2014	80,90%	(72,96% - 86,92%)	59234		89,56%	(83,35% - 93,63%)	29.744		82,56%	(69,82% - 90,65%)	25730	
2011 - 2012	87,32%	(79,08% - 92,62%)	56066		87,30%	(78,69% - 92,76%)	22.520		91,38%	(78,07% - 96,93%)	28808	
2010 - 2005	86,83%	(82,34% - 90,32%)	66376		88,06%	(82,75% - 91,90%)	48.202		81,28%	(66,87% - 90,34%)	9477	
2004 - 2000	77,40%	(68,08% - 84,61%)	25196		83,38%	(71,85% - 90,79%)	21.120		45,89%	(24,06% - 69,42%)	692	
Prior to 2000	73,69%	(67,91% - 78,76%)	36695		80,74%	(73,54% - 86,34%)	25.499		67,87%	(43,12% - 85,48%)	1952	
NR	71,16%	(64,75% - 76,83%)	84259		78,21%	(70,40% - 84,41%)	45.719		74,05%	(57,04% - 85,98%)	25617	
	**Non-consultation in case of illness or accident**											
	Total migrant population				Settled migrants				Emerging migrants			
2018 - 2017	9,44%	(3,40% - 23,60%)	1857	p-value: 0,2473	16,15%	(4,05% - 46,78%)	367	p-value: 0,3982	8,70%	(2,35% - 27,40%)	1418	p-value: 0,2951

2015 - 2016	13,42%	(6,88% - 24,54%)	3850		19,54%	(9,09% - 37,09%)	1113		10,84%	(4,43% - 24,21%)	2373	
2013 - 2014	10,37%	(4,06% - 24,02%)	895		9,22%	(2,21% - 31,35%)	347		11,63%	(2,81% - 37,50%)	474	
2011 - 2012	1,89%	(0,35% - 9,47%)	145		0,77%	(0,10% - 5,59%)	27		4,10%	(0,53% - 25,43%)	118	
2010 - 2005	8,95%	(3,76% - 19,83%)	1422		5,95%	(2,03% - 16,18%)	702		19,44%	(4,80% - 53,57%)	361	
2004 - 2000	7,24%	(2,34% - 20,23%)	532		8,83%	(2,79% - 24,60%)	521		0,00%	-	0	
Previo 2000	5,63%	(2,31% - 13,07%)	580		6,23%	(2,51% - 14,62%)	356		0,00%	-	0	
NR	7,99%	(4,63% - 13,47%)	1439		6,48%	(2,36% - 16,57%)	513		5,77%	(1,27% - 22,57%)	228	
	**Migrants without AUGE-GES treatment coverage**											
	Total migrant population				Settled migrants				Emerging migrants			
2018 - 2017	44,33%	(26,11% - 64,21%)	1832	p-value: 0,4914	42,96%	(20,33% - 68,97%)	174	p-value: 0,3982	45,33%	(24,03% - 68,49%)	1489	p-value: 0,1284
2015 - 2016	56,38%	(32,58% - 77,56%)	3209		67,76%	(24,45% - 93,18%)	1955		43,03%	(29,02% - 58,25%)	985	
2013 - 2014	41,38%	(16,43% - 71,70%)	1183		27,81%	(12,74% - 50,41%)	527		67,20%	(18,61% - 94,83%)	631	
2011 - 2012	35,43%	(8,33% - 76,82%)	818		11,50%	(1,35% - 55,31%)	161		0,00%	-	0	
2010 - 2005	46,74%	(29,39% - 64,91%)	2063		33,33%	(14,75% - 59,11%)	872		72,39%	(54,21% - 85,31%)	818	
2004 - 2000	30,00%	(16,49% - 48,18%)	1092		24,61%	(11,34% - 45,45%)	672		0,00%	-	0	
Prior to 2000	38,51%	(27,30% - 51,08%)	3722		44,89%	(28,18% - 62,84%)	2471		16,17%	(2,04% - 64,10%)	125	
NR	34,75%	(25,99% - 44,67%)	2959		23,55%	(15,16% - 34,69%)	932		26,89%	(10,37% - 53,89%)	235	
